# ASF1B: A Possible Prognostic Marker, Therapeutic Target, and Predictor of Immunotherapy in Male Thyroid Carcinoma

**DOI:** 10.3389/fonc.2022.678025

**Published:** 2022-01-31

**Authors:** Weigang Qiu, Xinquan Wu, Haihong Shi, Bingyang Liu, Liqiong Li, Wenyi Wu, Jianqing Lin

**Affiliations:** Department of Thyroid and Breast Surgery, The Second Affiliated Hospital of Fujian Medical University, Quanzhou, China

**Keywords:** male thyroid cancer, hub genes, ASF1B, FOXP3, Treg, tumor immunity

## Abstract

**Background:**

Thyroid carcinoma (TC) is the most common malignant endocrine tumor worldwide. Several studies have documented that male patients with TC have a higher rate of metastasis and disease recurrence than female patients. However, the mechanism underlying this observation is not completely clear. The goal of our research was to investigate the potential key candidate genes and pathways related to TC progression in male patients at the molecular level.

**Methods:**

A total of 320 samples were obtained from The Cancer Genome Atlas (TCGA) and Genotype-Tissue Expression (GTEx) databases. Hub genes were screened out using weighted gene coexpression network analysis (WGCNA) and a protein–protein interaction (PPI) network analysis. Survival analysis was used to identify hub genes associated with disease-free survival (DFS) rates. Estimation of STromal and Immune cells in MAlignant Tumor tissues using Expression (ESTIMATE) data were used to assess the relationship between hub genes and immune cell infiltration. The molecular mechanism and biological functions of hub genes were explored using RT-qPCR, Western blot, Cell Counting Kit-8 Assay, flow cytometry, Transwell assays, and scratch assays.

**Results:**

Forty-seven hub genes were identified, and the survival analysis demonstrated that anti-silencing function 1B (ASF1B) was the sole independent risk factor for poor DFS in male TC patients. Possible associations between the results from the ESTIMATE analysis showed that the ASF1B expression level was related to the ESTIMATE score, immune score, and T-cell regulatory (Treg) infiltration level. Through *in vitro* cell function experiments, we verified that knockdown of ASF1B inhibited KTC-1 cell proliferation, promoted cell apoptosis, and blocked cell cycle. The silencing of ASF1B reduced protein kinase B (AKT), phospho-AKT (p-AKT), and forkhead box p3 (FOXP3) in KTC-1 cells. Moreover, FOXP3 overexpression markedly restored the cell migration, invasion, and proliferation abilities repressed by ASF1B knockdown.

**Conclusions:**

Our results indicate that ASF1B can be considered a prognostic marker, therapeutic target, and predictor of immunotherapy response in male thyroid cancer patients. However, further in-depth studies are required to validate this finding.

## Introduction

Thyroid carcinoma (TC) is the most common malignant endocrine tumor worldwide (1, 2). According to the 2018 Global Cancer Statistics, TC accounted for 2% of all cancers and 41,000 deaths that year. Although TC is three times more common in women than in men (3), male sex was found to be associated with a poor TC prognosis in multiple studies (4–6). The mechanism responsible for this phenomenon is unclear. In some studies, the authors have suggested that female hormonal and reproductive processes might be implicated in the growth and aggressive behavior of TC cells (7–9). However, the above result could not explain opposite conclusions drawn from another study of female patients in Los Angeles county (10). More research is needed to understand the unique features of TC in males.

Hub genes are a class of genes that play paramount roles in many cellular physiological processes. In any pathway, a small number of hub genes often influence the expression of other genes (11). The development of TC is a complex process resulting from a substantial number of genetic and epigenetic alterations (12). Many genetic alterations have been identified and play fundamental roles in TC development and progression, such as mutations in BRAF, RAS, and RET (13–16). However, Hub genes unique to TC in males have not been previously reported.

The immune system plays an important role in the prevention and progression of malignancy (17). There is increasingly strong evidence for the robust association between inflammation and TC (18). So far, however, there has been little discussion about the immune marker of TC in males.

In our study, we explored the key role of ASF1B in male TC through bioinformatics analyses and survival analysis. The data used in our study were obtained from TCGA, GTEx, and the Gene Expression Omnibus (GEO) database (19). In addition, we employed TISIDB (20), the CIBERSORT webserver tool (21, 22), and ESTIMATE (23) to explore the relationship between ASF1B expression level and immune infiltration in TC in males.

Previous studies demonstrated that the PI3K/Akt signaling pathway and FOXP3 is an attractive therapeutic target for TC (24–26). P-AKT and AKT serve as the core factor in the PI3K/AKT signaling pathway (27). FOXP3, which is related to tumor immune microenvironment and immune escape, is also one of hub genes in male TC (28, 29). In this study, we verified that the silencing of ASF1B reduced p-AKT, AKT, and FOXP3 in KTC-1 cells. Knockdown of ASF1B inhibited KTC-1 cell proliferation, promoted cell apoptosis, and blocked cell cycle. Moreover, FOXP3 overexpression markedly restored the cell migration, invasion, and proliferation abilities repressed by ASF1B knockdown. Based on these findings, we suggest that as a hub gene, ASF1B is closely correlated with the proliferation, migration, and invasion of male TC cells. In addition, ASF1B may mediate Treg cells recruitment by regulating the expression of FOXP3. This may be one of the mechanisms of the immune escape of male TC cells. Collectively, our results identify ASF1B as a novel potential therapeutic target that is associated with the progression of male TC.

## Materials and Methods

### Male TC RNA-Seq Dataset and Clinical Information

The data used in this paper were obtained from two main sources, the TCGA and GTEx databases. All transcript RNA-seq datasets of tissues were downloaded from the University of California Santa Cruz (UCSC) Xena database (30). Transcript expression levels were estimated using the FPKM (fragments per kilobase per million fragments mapped) method in HTSeq. A total of 320 samples (130 tumor tissues and 190 normal tissues) were included in our study. All specimens were taken from the thyroid. Data on 130 tumor tissues and 17 normal tissues were obtained from the TCGA database, and data on 173 normal tissues were obtained from the GTEx database. Clinical and follow-up data for each tumor sample (*n* = 130) were obtained from the TCGA data portal (up to Jan 28, 2016). The data are summarized in **Supplementary Table 1**.

### Data Preprocessing

Batch correction was performed on the RNA-seq datasets from different sources. Batch correction was performed using the Combat method of the sva package (version 3.36.0) in R. Before batch correction, the normalization of the GTEx data was adjusted from log2(x+0.001) to log2(x+1) to align with the normalization of the TCGA data.

### Screening of Differentially Expressed Genes

The limma package (version 3.44.1) was used to identify DEGs (31). |Fold Change (FC)| > 2 and *p*-value < 0.05 after FDR (false discovery rate) adjustment were established as cutoff criteria. Heatmaps of DEGs were generated using the R package pheatmap (version 1.0.12).

### Construction of Gene Coexpression Network

Weighted gene coexpression network analysis (WCGNA) was used to construct a coexpression network based on scale-free topology to detect coexpression modules of genes with similar patterns of expression (32). In our study, DEGs were input into the WGCNA package (v1.69) in R to construct weighted coexpression modules. The module membership (kME) value was used to weight the importance of a gene in a module (33). In our study, DEGs with |kME| values > 0.8 were considered hub genes of the weighted gene coexpression network (34). The relationships between modules and clinical traits were confirmed by Pearson’s correlation and are shown in a heatmap. The following clinical traits were analyzed: T, tumor stage, divided into two groups according to the 8th American Joint Committee on Cancer (AJCC) editions (T1–2 and T3–4); N, lymph node metastasis stage, divided into two groups according to the 8th AJCC editions (N0/Nx and N1); M, distant metastasis stage, divided into two groups according to the 8th AJCC editions: (M0/Mx and M1); stage, cancer stage, divided into two groups according to the 8th AJCC editions (stage I–II and stage III–IV); age, divided into two groups (≤60 years of age and >60 years of age); and extent of the primary tumor. In thyroid surgical practice, the thyroid is divided into three anatomical zones, the left lobe, right lobe, and thyroid isthmus. Patients were divided into two groups according to postoperative pathological findings depending on whether they had tumors confined to a single lobe or tumors in multiple lobes. A *p*-value < 0.05 was considered significant.

### Construction of Protein–Protein Interaction Network

Protein–protein interaction (PPI) analysis is useful for tracking potential interactions among DEGs. We used the STRING online database (version 11.0) to construct a PPI network of the DEGs (35, 36). Degree indicates the number of connections/interactions involving the hub genes. To screen the hub genes of the PPI network, the selection criterion for nodes was set as degree ≥10 (37).

### Functional Analyses of Modules Containing Hub Genes

The intersection of hub genes of the weighted gene coexpression network and PPI network were regarded as hub genes of TC in males. A network of the 47 overlapping hub genes and 13 modules containing these hub genes was constructed by Cytoscape (version 3.8.0). Genes within the same module were analyzed for enrichment of Gene Ontology (GO) terms and Kyoto Encyclopedia of Genes and Genomes (KEGG) pathways using DAVID bioinformatics resources (version 6.8) (38, 39). *p*-values <0.05 and counts >3 were considered threshold values (40).

### Survival Analysis Based on Hub Genes

Survival analyses were performed using the survival package in R (survival package version 3.1-8). Patients were split into two groups (low expression/high expression) according to the expression level of each hub gene. The Kaplan–Meier method and a log-rank test were used to compare the DFS rates between the two groups. Additionally, univariate Cox proportional hazard regression analysis was performed for each hub gene to test its association with the DFS rate of male TC patients. Hub genes with *p*-values less than 0.05 in the Kaplan–Meier survival curve and univariate Cox analysis were entered into a multivariate Cox proportional hazards model. A *p*-value <0.05 was set as the threshold value. Finally, ASF1B expression was screened using the method described above. Then, a ROC curve was used to establish the best cutoff point for ASF1B levels that optimally predicted the DFS rate. The best cutoff value for the predictive equation was achieved when the Youden index (YI = sensitivity + specificity − 1) was at its maximum. ASF1B high/low expression groups were determined according to the best cutoff value. Finally, the predictive power of ASF1B expression levels and clinical features in combination was analyzed using multivariate Cox regression. *p* < 0.05 was used as the criterion for statistical significance. The area under the curve (AUC) values and ROC curves were calculated using SIGMAPLOT (version 14, Systat Software INC, CA, USA).

### Mining the Immune-Related Mechanism of ASF1B

To analyze the relationship between the tumor immune microenvironment and the expression levels of ASF1B, TISIDB online tools, the CIBERSORT algorithm, and the ESTIMATE algorithm were used. TISIDB is a database for tumor and immune system interactions (41). We evaluated the difference in the ASF1B expression level between responders and nonresponders to anti-PD-L1 (atezolizumab) therapy using TISIDB online tools. The data used for this analysis were obtained from a previously published urothelial cancer research study (42).

We further explored the relationship between the ASF1B expression level and tumor immune cell infiltration in TC in males using the CIBERSORT tool. CIBERSORT is a computational inference tool that can estimate the abundance of 22 leucocyte subtypes in tumors based on RNA-seq data. Each tumor sample was scored by the “CIBERSORT” R package (version 1.0.3).

In this study, correlations between ASF1B expression levels and infiltration level/ESTIMATE scores were calculated using the Spearman method. For consistency with the DEG results, the ASF1B expression levels were normalized to FPKM log2(x+1) format for the Spearman correlation analysis. The R package “ESTIMATE” (version 1.0.13) was used for this analysis, and a *p*-value <0.05 was set as the threshold value.

### Validation of ASF1B Differential Expression in TC in Males Using Data From the GEO Database

To verify the difference in the ASF1B expression level between male TC tissues and normal male thyroid tissues, we downloaded relevant data from the GEO database. The validation set consisted of 96 samples from male TC patients (71 tumor tissues and 25 normal tissues). Gene expression profiling data were taken from the GSE-29265, GSE-3467, and GSE-76039 datasets. These data were quantified using the metric log2(FPKM) and then corrected for batch effects using the Combat method of the R package sva (version 3.36.0). The differences in ASF1B expression levels between tumor tissues and normal tissues were compared by independent-samples *t*-tests in R software (version 3.6.3). A *p*-value <0.05 was set as the threshold. Violin plots were utilized to visualize the distribution of ASF1B expression levels among samples.

### Cell Culture and Transfection

The human thyroid cell line (KTC-1) was purchased from Zolgene Technology Co., Ltd. (Zolgene, China). The KTC-1 cell line was originally derived from a 68-year-old white male TC patient (43). This cell line is consistent with the main characteristics of the samples in our study. KTC-1 cells were cultured in RPMI 1640 (Meilune Biotech, China) supplemented with 10% fetal bovine serum (FBS), 100 U/ml penicillin, and 100 μg/ml streptomycin. All cells were maintained in a humidified incubator with 5% CO_2_ at 37°C.

Short hairpin RNA (shRNA) targeting ASF1B and scrambled control shRNA were designed and synthesized by Anburui Co., Ltd. (Anburui, China). All shRNA sequences used in this study are provided in **Supplementary Table 6**. KTC-1 cells were seeded at 1 × 10^4^ cells/well into 6-well plates in complete media. The shRNAs and the shRNA-negative control vector were purchased from Shanghai Yusheng Biotechnology (Shanghai, China). These vectors were introduced into cells using Hieff Trans™ liposomal transfection reagent following the manufacturer’s protocol. The cells were washed and collected for subsequent experiments after incubation at 37°C for 12 h.

ASF1B knockdown levels were measured in KTC-1 cells transfected with 3 different shRNAs targeting ASF1B (sh1, sh2, and sh3). A blank group (Blank) and a negative group (shRNA-NC) were included as controls.

### RNA Extraction and Reverse Transcription-Quantitative Polymerase Chain Reaction

RNA was extracted from cells using RNAiso Plus (Takara, China). A total of 1 µg of RNA was transcribed to cDNA using NovoScript^®^ Plus All-in-one 1st Strand cDNA Synthesis SuperMix (gDNA Purge) Novoprotein, E047-01A (Shanghai, China). The resulting RT product was amplified using NovoStart^®^ SYBR qPCR Supermix (Novoprotein, China), and the data were collected using an iQ5 Real-Time PCR Detection System (Bio-Rad, USA). Glyceraldehyde-3-phosphate dehydrogenase (GAPDH) was used as the loading control. The relative gene expression was generated using the 2^−ΔΔCT^ method (ΔCT target gene − ΔCT control gene).

### Cell Counting Kit-8 Assay

Cellular proliferation was determined by CCK-8 following the manufacturer’s instructions (Meilune, China). Cell suspensions (100 µl, 5,000 cells) of sh-ASF1B and sh-ASF1B NC cells were added to each well of a 96-well plate. After cell culture for 0 h, 24 h, 48 h, and 72 h, respectively, 10 μl of CCK-8 solution was added to each well and incubated at 37°C for 1 h. The absorbance was measured at a wavelength of 450 nm for each well.

### Flow Cytometry Analysis

We analyzed the apoptosis and cell cycle of KTC-1 cell lines using Flow Cytometry. After indicated treatments, for the cell apoptosis analysis, KTC-1 cells were collected separately, and incubated with Annexin-V (Meilune, China) in the dark for 15 min, and the apoptotic cells were analyzed using a FACSCalibur (BD, USA). For the cell cycle analysis, KTC-1 cells were collected separately, resuspended in 1 ml of ice-cold ethanol 75% overnight at 4°C, incubated with RNaseA (Meilune, China) at 37°C for 30 min, incubated with propidium iodide (PI) in the dark at 4°C for 30 min, and then detected by FACSCalibur (BD, USA).

### Transwell Invasion Assay

The Transwell invasion assay was performed using a Transwell chamber with a Matrigel-coated filter (Corning, USA). First, cells were suspended in serum-free medium. Then, the cells were added to the upper chamber and cultured with serum-free DMEM, whereas the lower chamber was filled with complete medium supplemented with 20% fetal bovine serum (Gibco, USA) and 1% penicillin and streptomycin (HyClone, UK). After being cultured in a 24-well plate at 37°C and 5% CO_2_ for 24 h, the cells were blocked with 4% paraformaldehyde fixative and dyed with crystal violet (20% methanol, 0.1% crystal violet in H_2_O). Finally, the invasive cells were counted in three random fields under a microscope. The experiment was repeated three times.

### Wound Healing Analysis

The KTC-1 cells were selected, digested and counted, and then inoculated into 6-well culture plates. Scratch was made by a 200-μl pipette tip when the cell confluence grew to 90%. Select the same point of view to take a picture at 0 h and 12 h. The wound width was monitored by an inverted microscope for assessing the capacity of KTC-1 cell migration.

### Western Blotting

Protein cells were collected using RIPA lysis buffer (KGP702, KeyGen Biotech, China). Protein concentrations were determined using a Pierce BCA Protein Assay Kit (Thermo, No. 23227, USA). SDS-PAGE (10%) was applied to isolate proteins, which were later transferred onto PVDF membranes. Membranes were blocked and incubated with primary antibodies overnight in nonfat milk at 4°C. Secondary antibody was applied at RT for 1–1.5 h. Signal detection was performed with chemiluminescence imaging system (Bio-Rad, USA).

### FOXP3 Overexpression Rescue Experiments

A rescue experiment was performed by overexpressing FOXP3 in ASF1B-silenced KTC-1 cells. Cells were transfected with FOXP3-overexpressing plasmid (zolgene Biotech, China; Plasmid sequences are listed in **Supplementary Table 7**) or empty plasmid using Lipofectamine3000 kit (Invitrogen, USA) according to the manufacturer’s instruction. Cells were harvested at 48–96 h after transfection and RT-qPCR was used to detect mRNA expression. The effects of FOXP3 overexpression on proliferation and migration of ASF1B-silenced KTC-1 cell lines were examined by CCK8 assay and scratch assay; the detailed experimental scheme is the same as before.

## Results

### Data Preprocessing and Identification of DEGs in Male TC

**Figure 1:**
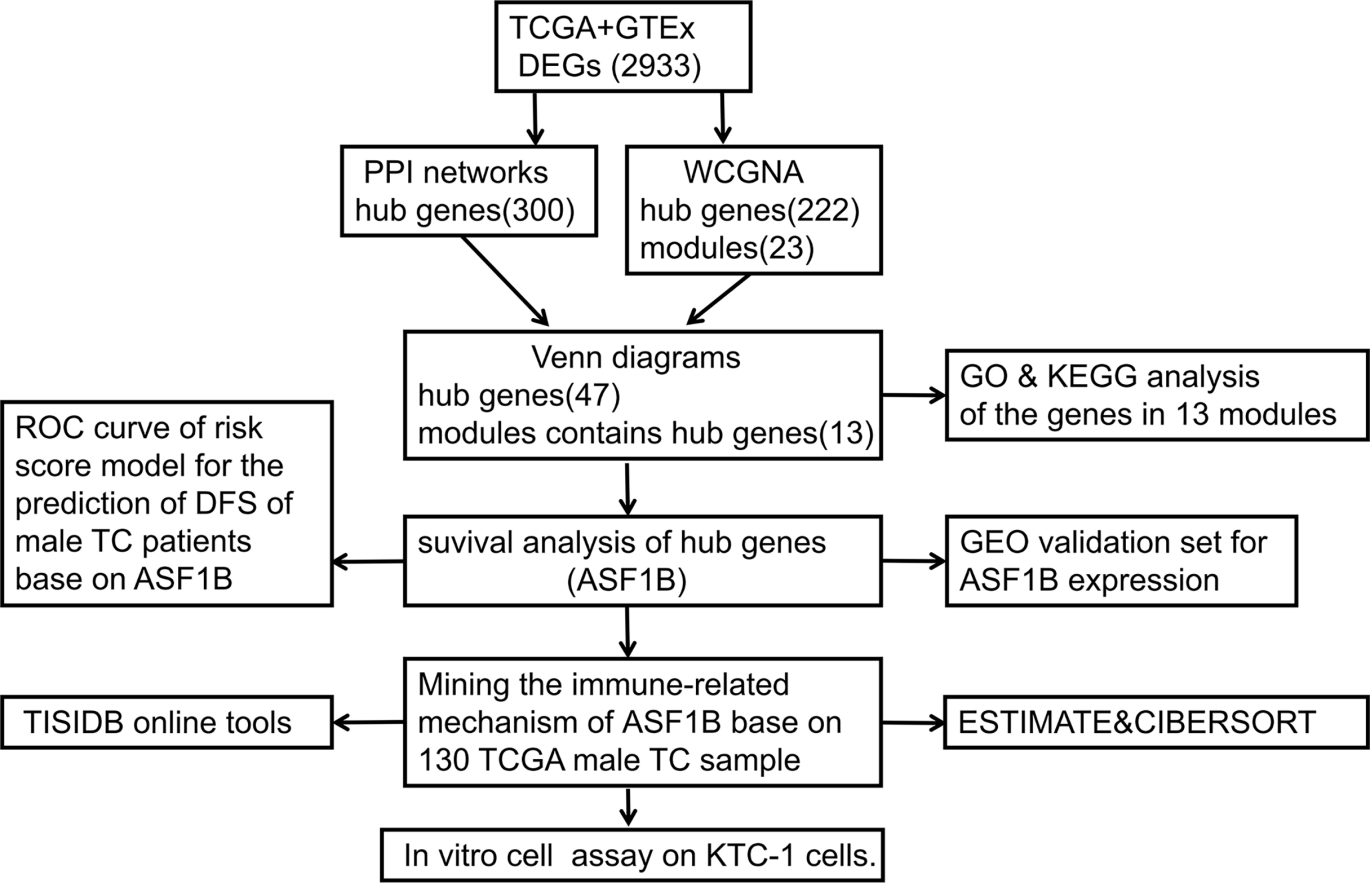
Flowchart of the study process.

**Figure 2:**
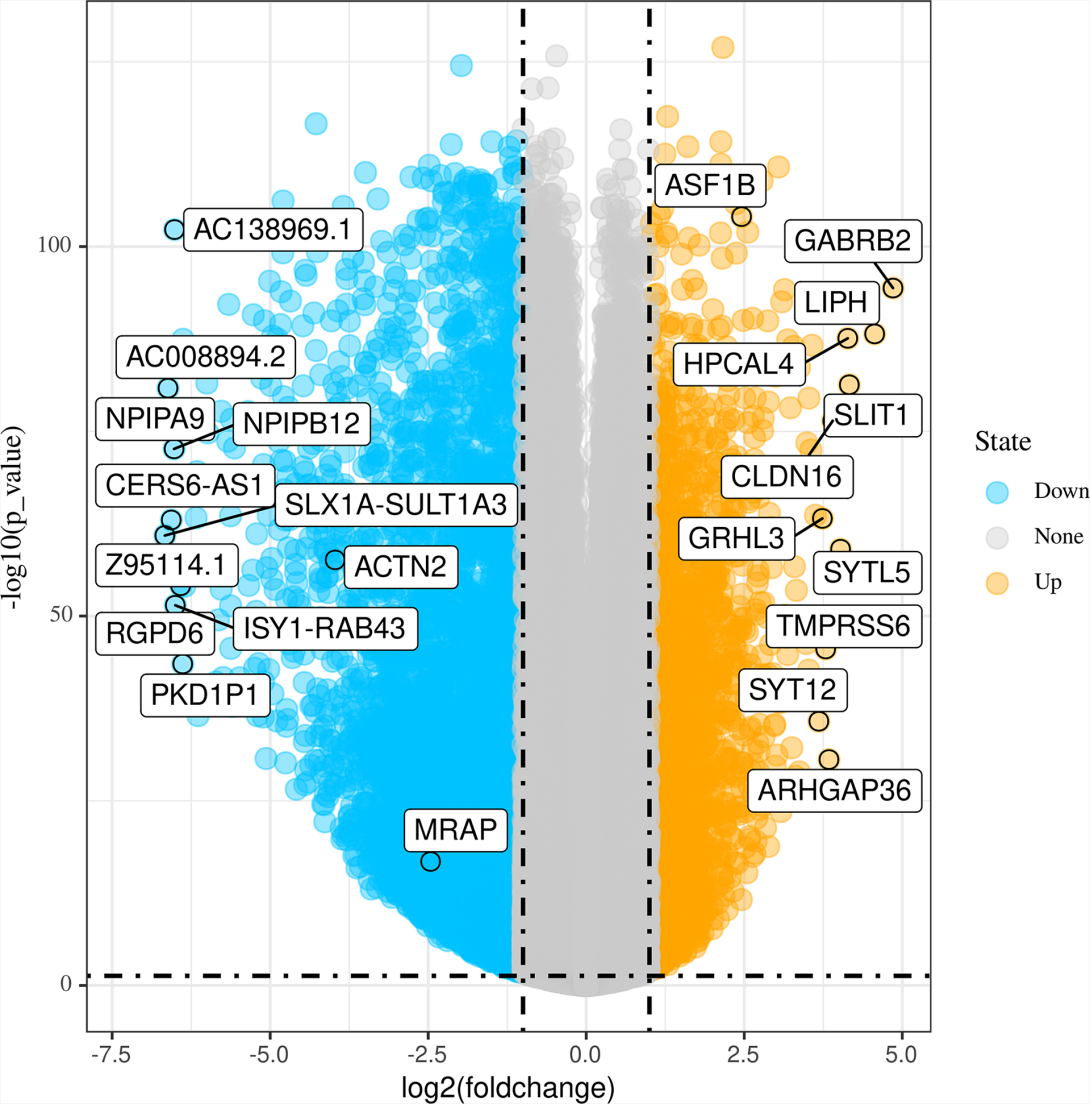
Volcano plots of differentially expressed genes (DEGs) between tumor tissues and normal tissues.

**Figure 3:**
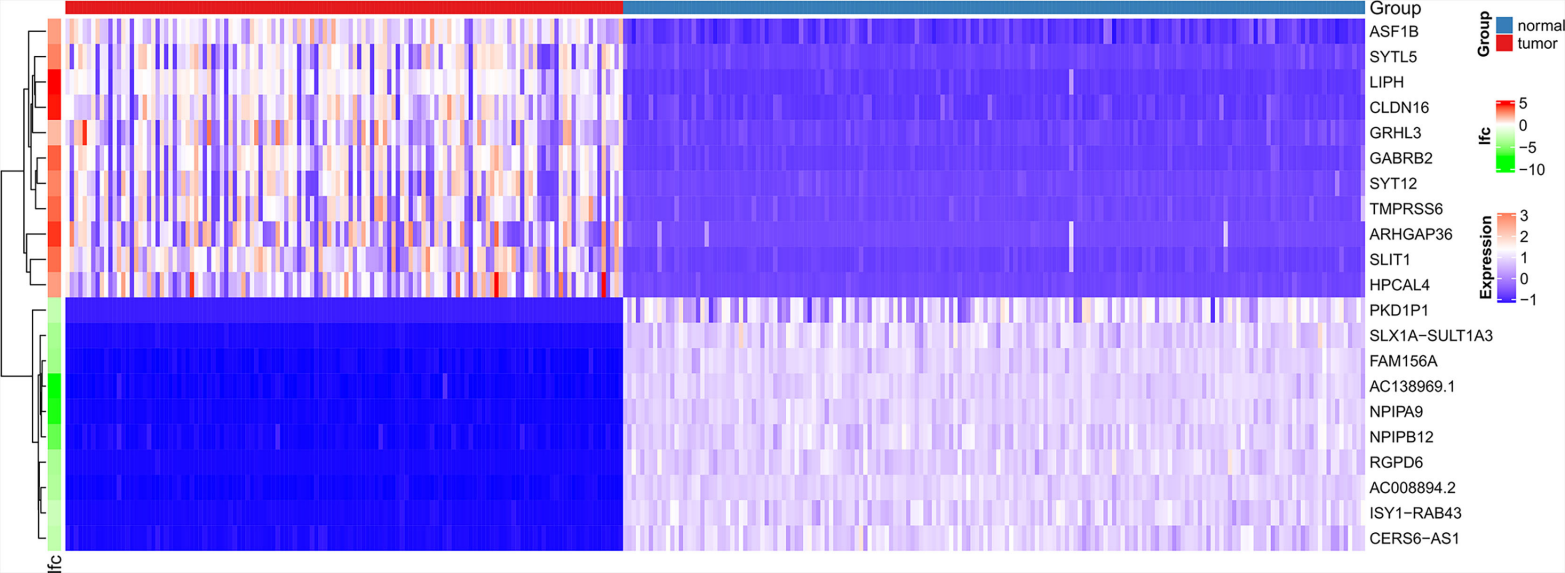
Heatmaps of differentially expressed genes. The top 20 dysregulated (10 upregulated and 10 downregulated) DEGs and ASF1B are shown in a heatmap.

**Figure 1** shows the workflow of our research. Since the microarray data were collected from two different sources, data were first subjected to batch correction before differential gene expression analyses (**Supplementary Figure 1**). A total of 2,933 genes were differentially expressed, with 331 upregulated genes and 2,602 downregulated genes (**Figure 2** and **Supplementary Table 2**). The heatmap represented the top 10 upregulated DEGs and top 10 downregulated DEGs between tumor tissues and normal tissues (**Figure 3**).

### Hub Genes and Key Modules Generated by WCGNA

**Figure 4:**
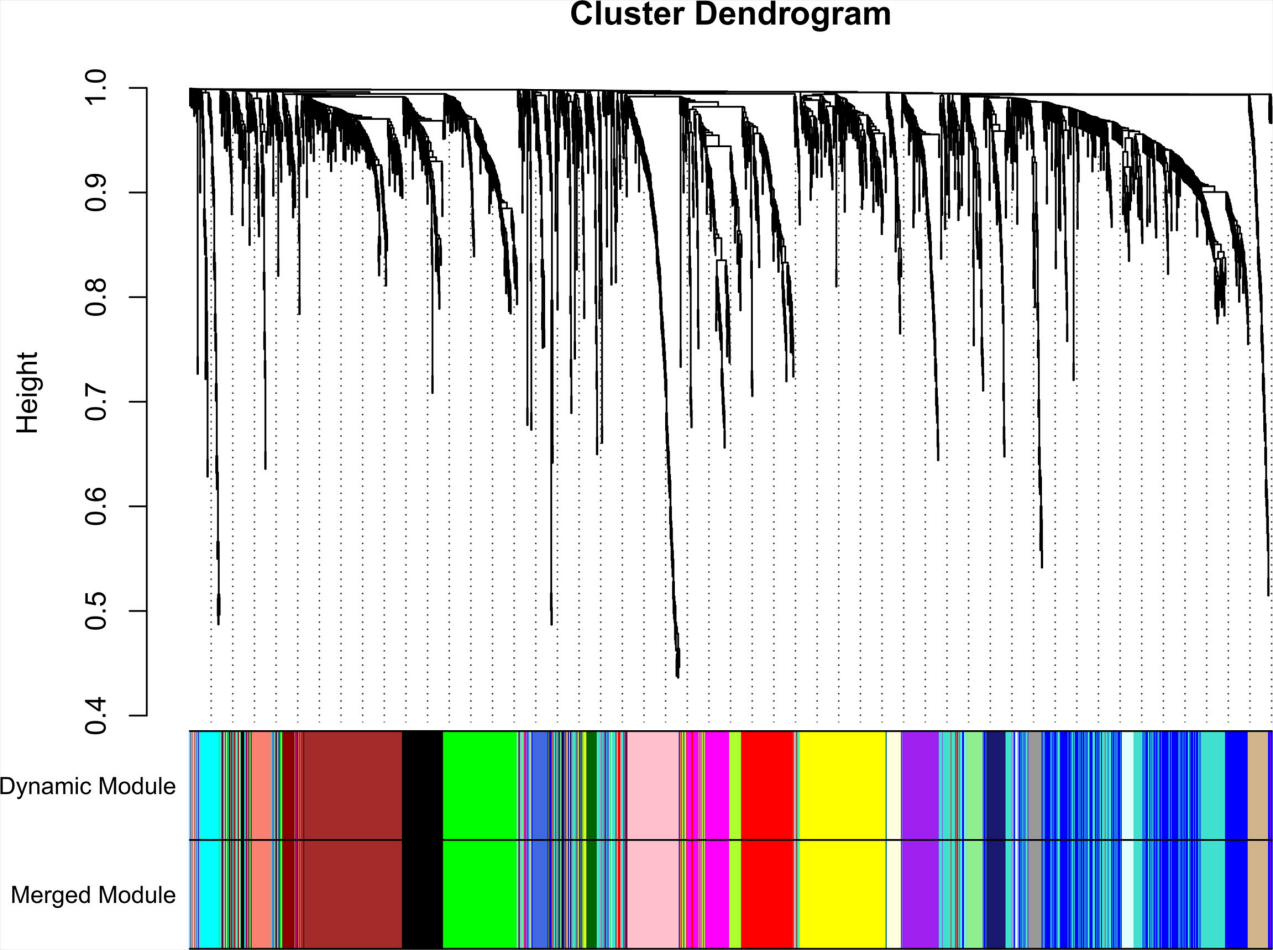
Sample clustering indicated that no outliers were present that required removal from the subsequent analysis.

**Figure 5:**
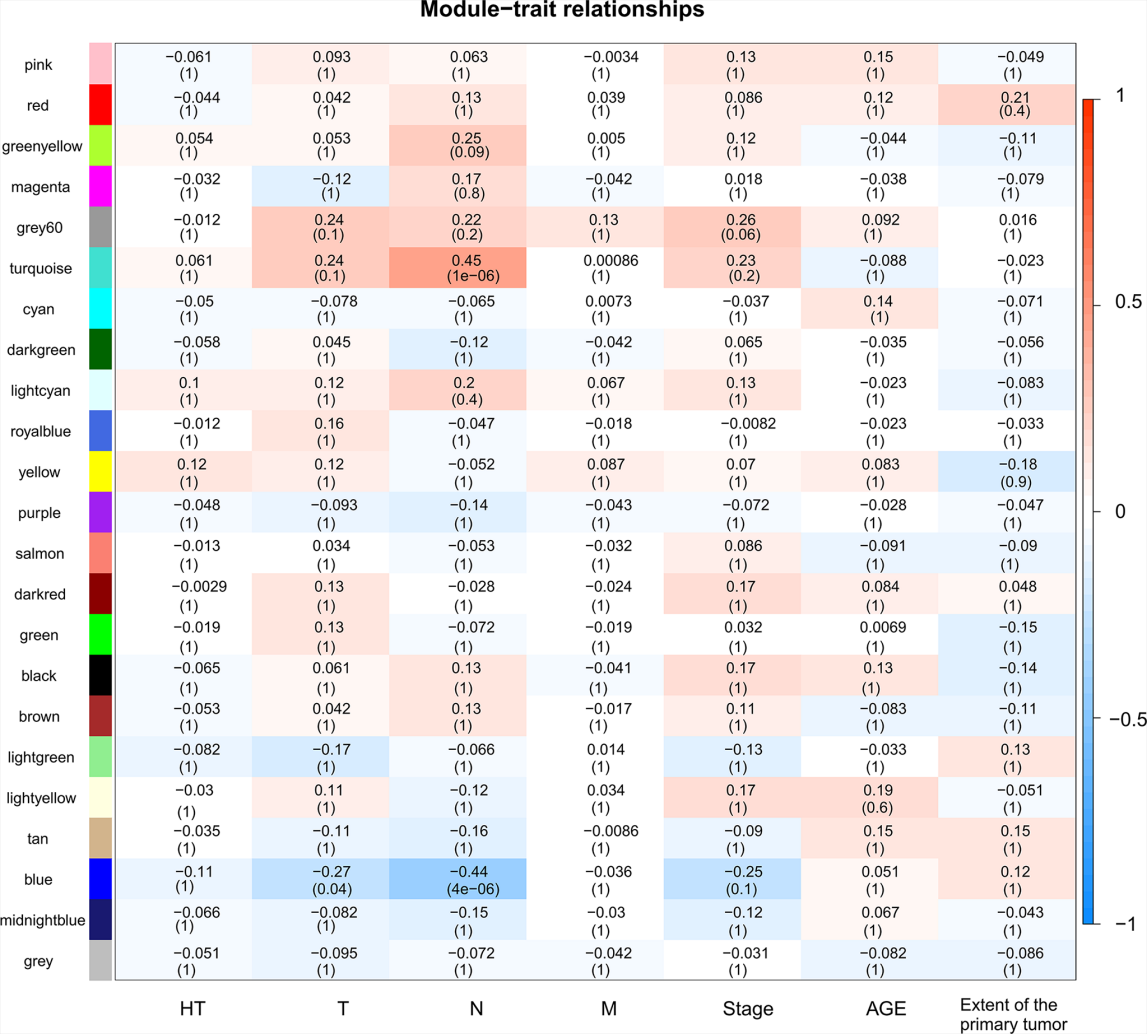
Heatmap of module-trait relationships. Each row corresponds to a module, and each column corresponds to a trait. The progressively increase in blue and red saturation indicated a high Pearson’s correlation coefficient. The numbers in the squares indicate Pearson’s correlation coefficients.

A total of 130 samples with clinical data available and 2,933 DEGs were included in the WCGNA. Significant abnormal values were not found in the hierarchical clustering results (**Supplementary Figure 2A**). To ensure a scale-free network, soft-threshold analysis was performed on all samples. We chose a soft thresholding power (*β*) of 4, and this value fitted the scale-free topology assumptions. The linear regression model fitting index R2 was greater than 0.8. The mean connectivity was almost 0 when *β* = 4 (**Supplementary Figures 2B, C**). Using the dynamic tree cut and merging similar modules method, a total of 23 modules were identified (**Figure 4**). A heatmap shows the relationships between the clinical traits and each module by Pearson’s correlation. The turquoise module (*r* = 0.45, *p* = 1e-6) and blue module (*r* = −0.44, *p* = 4e-6) were strongly correlated with lymphatic metastasis; blue modules (*r* = −0.27, *p* = 0.04) were strongly correlated with T stage (**Figure 5**). |kME| value > 0.8 was used as a screening standard for the selection of hub genes and identified 222 hub genes among the DEGs (**Supplementary Table 3**).

### Identifying Hub Genes of PPI Networks

We constructed a PPI network of 2,933 DEGs using the STRING database. A total of 300 genes with a degree ≥10 were selected from the PPI network as hub genes (**Supplementary Table 4**).

### Functional Analyses of Modules Containing Hub Genes

**Figure 6:**
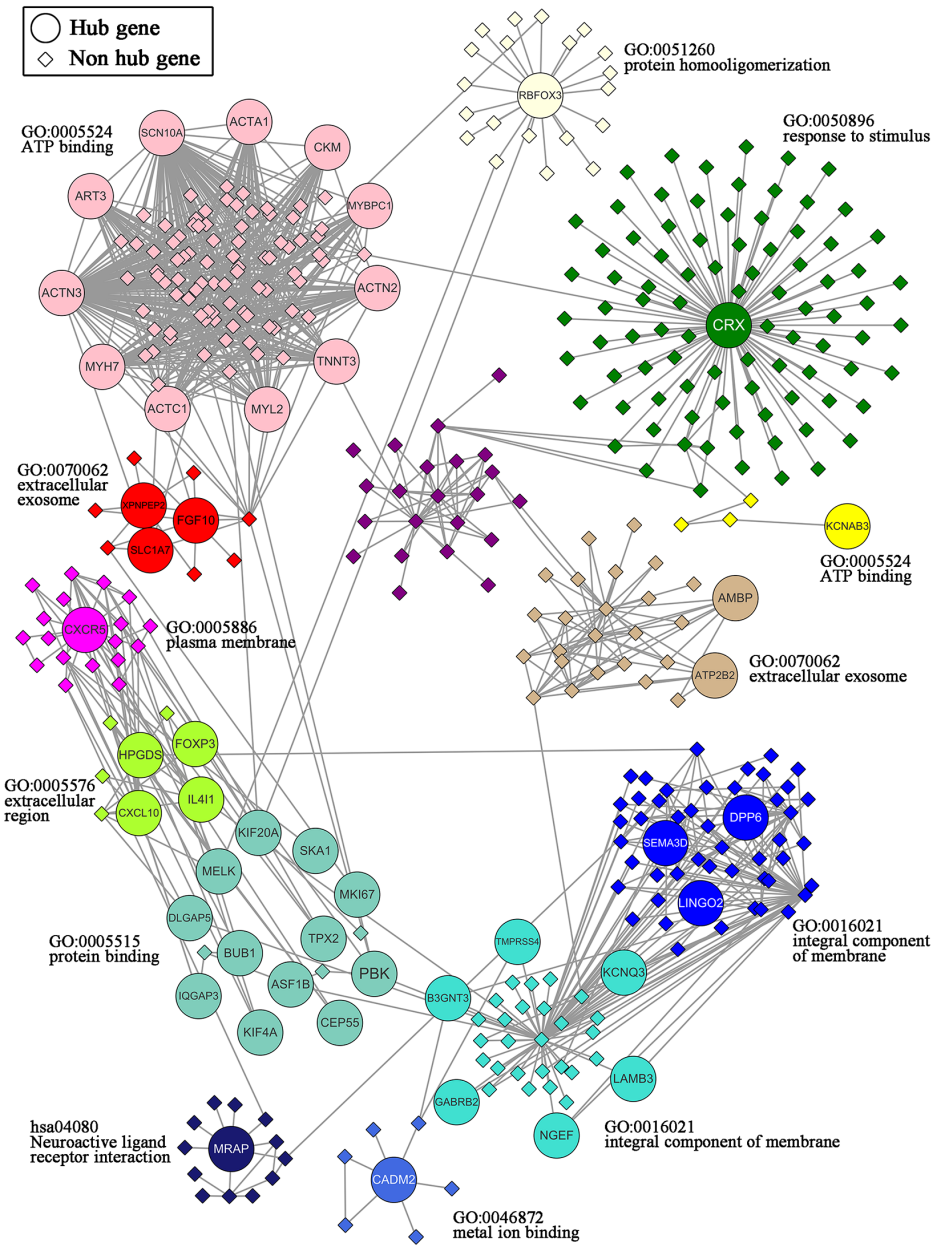
Network of the 47 hub genes and the 13 modules containing these hub genes. 47 hub genes: ACTA1, ACTC1, ACTN2, ACTN3, AMBP, ART3, ASF1B, ATP2B2, B3GNT3, BUB1, CADM2, CEP55, CKM, CRX, CXCL10, CXCR5, DLGAP5, DPP6, FGF10, FOXP3, GABRB2, HPGDS, IL4I1, IQGAP3, KCNAB3, KCNQ3, KIF20A, KIF4A, LAMB3, LINGO2, MELK, MKI67, MRAP, MYBPC1, MYH7, MYL2, NGEF, PBK, RBFOX3, SCN10A, SEMA3D, SKA1, SLC1A7, TMPRSS4, TNNT3, TPX2, XPNPEP2.

A total of 47 overlapping hub genes between the WGCNA and PPI networks were identified using the Venn diagrams online tool (44) (**Supplementary Figure 3**). Forty-seven hub genes were distributed across 13 modules (**Figure 6**). Gene ontology (GO) and KEGG enrichment analyses were conducted for the genes in each module, and the gene sets of 9 modules showed enrichment in GO or KEGG pathways (**Supplementary Figure 4**). The most significant pathway for each module is indicated in the text label near each module in **Figure 6**. Different colors represent different modules and the genes they contain.

### Survival Analysis of Hub Genes

**Figure 7:**
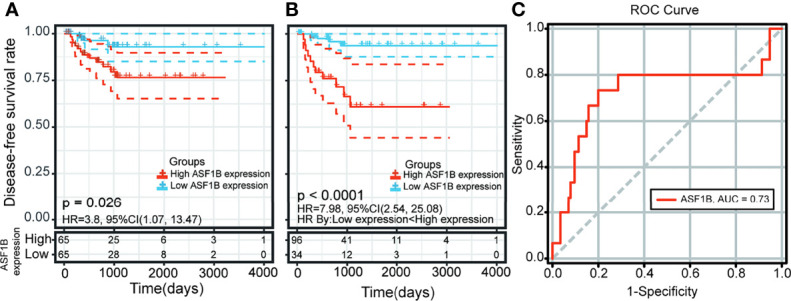
Kaplan-Meier curve and receiver operating characteristic (ROC) curve analysis of disease-free survival (DFS) in male thyroid carcinoma patients based on ASF1B expression levels. **(A)** Patients were divided into low- and high-expression groups according to the median value of ASF1B expression. **(B)** Patients were divided into low- and high-expression groups according to the best cutoff points (2.96) determined by the ROC curve analysis. **(C)** Area under the ROC curve (AUC) of ASF1B expression in male thyroid carcinoma patient tumors.

Kaplan–Meier survival and univariate Cox analysis showed that a high ASF1B expression level was a risk factor for DFS (the patients were grouped by median ASF1B expression) (**Figure 7A** and **Supplementary Table 5**). The best cutoff point of ASF1B expression (2.96) was calculated according to the maximum Youden index in the ROC curve for the DFS rate. The sensitivity and specificity were 73.3% [95% confidence interval (CI) 0.45, 0.92] and 80.0% (95% CI 0.72, 0.87), respectively, at the best cutoff point. The AUC had a value of 0.73 (95% CI 0.55–0.90) (**Figure 7C**). Multivariate survival analysis showed that the ASF1B expression level was the sole independent risk factor for the DFS rate when the ASF1B high/low expression groups were divided according to the best cutoff points determined by the ROC curve analysis [hazard ratio (HR) 7.98; 95% CI 2.54, 25.8] (**Figure 7B** and **Table 1**).

**Table 1:** Multivariate Cox regression analysis in male TC patients.

	Multivariate analysis	
	*p*-value	HR (95% CI)
ASF1B (High expression level vs. Low expression level)*	0.001	7.823 (2.266–27.013)
Age (>60 vs. ≤60)	0.928	0.943 (0.266–3.339)
Histological type (NON PTC vs. PTC)**	0.988	0.001 (0.001–∞)
Tumor size (T3–4 vs. T1–2)	0.552	1.481 (0.406–5.406)
Lymph node status (N1 vs. N0&Nx)	0.447	0.637 (0.201–2.034)
Metastasis (M1 vs. M0&Mx)	0.629	1.717 (0.192–15.361)
Pathological stage (S3–4 vs. S1–2)	0.098	3.666 (0.787–17.072)
Extent of the primary tumor (Single lobectomy vs. Multiple lobectomies)***	0.451	6.937 (0.449–6.038)

Tumor stage (T), lymph node metastasis stage (N), and metastasis (M) were divided into two groups according to the 8th American Joint Committee on Cancer (AJCC) editions. *Grouped according to the best cutoff points determined by the ROC curve. **PTC, Papillary thyroid carcinoma. ***In thyroid surgical practice, the thyroid is divided into three anatomical zones, the left lobe, right lobe, and thyroid isthmus.

### The Association Between ASF1B and Immune Cell Infiltration

**Figure 8:**
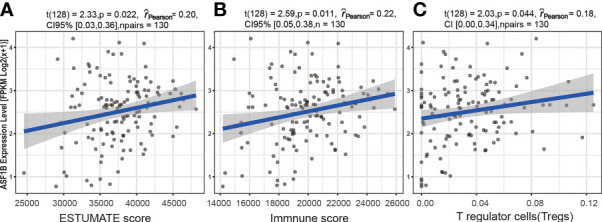
The association between the ASF1B expression level and immune cell infiltration. Relationship between the ESTIMATE score and ASF1B expression level **(A)**, between the immune score and ASF1B expression level **(B)**, and between T regulatory cells (Tregs) and the ASF1B expression level **(C)**.

A significant correlation was detected between the ASF1B expression level [fpkm log2(x+1)] and six immune factors. Spearman rank analyses demonstrated that ASF1B expression levels were significantly positively correlated with the ESTIMATE score (ρSpearman = 0.20, *p* < 0.05, 95% CI 0.03, 0.36), immune score (ρSpearman = 0.20, *p* < 0.05, 95% CI 0.03, 0.36), and T regulatory cell (Treg) infiltration level (ρSpearman = 0.22, *p* < 0.05, 95% CI 0.05, 0.38) (**Figure 8**). In addition, the results based on the analysis of previously published urothelial cancer research showed that patients with high expression of ASF1B would not respond to anti-PD-L1 (atezolizumab) therapy (230 nonresponders vs. 68 responders, log2FC = 0.436, *p* < 0.01) (42).

### Validation of the Aberrant Expression of ASF1B in TC Samples From Male Patients in the GEO Database

**Figure 9:**
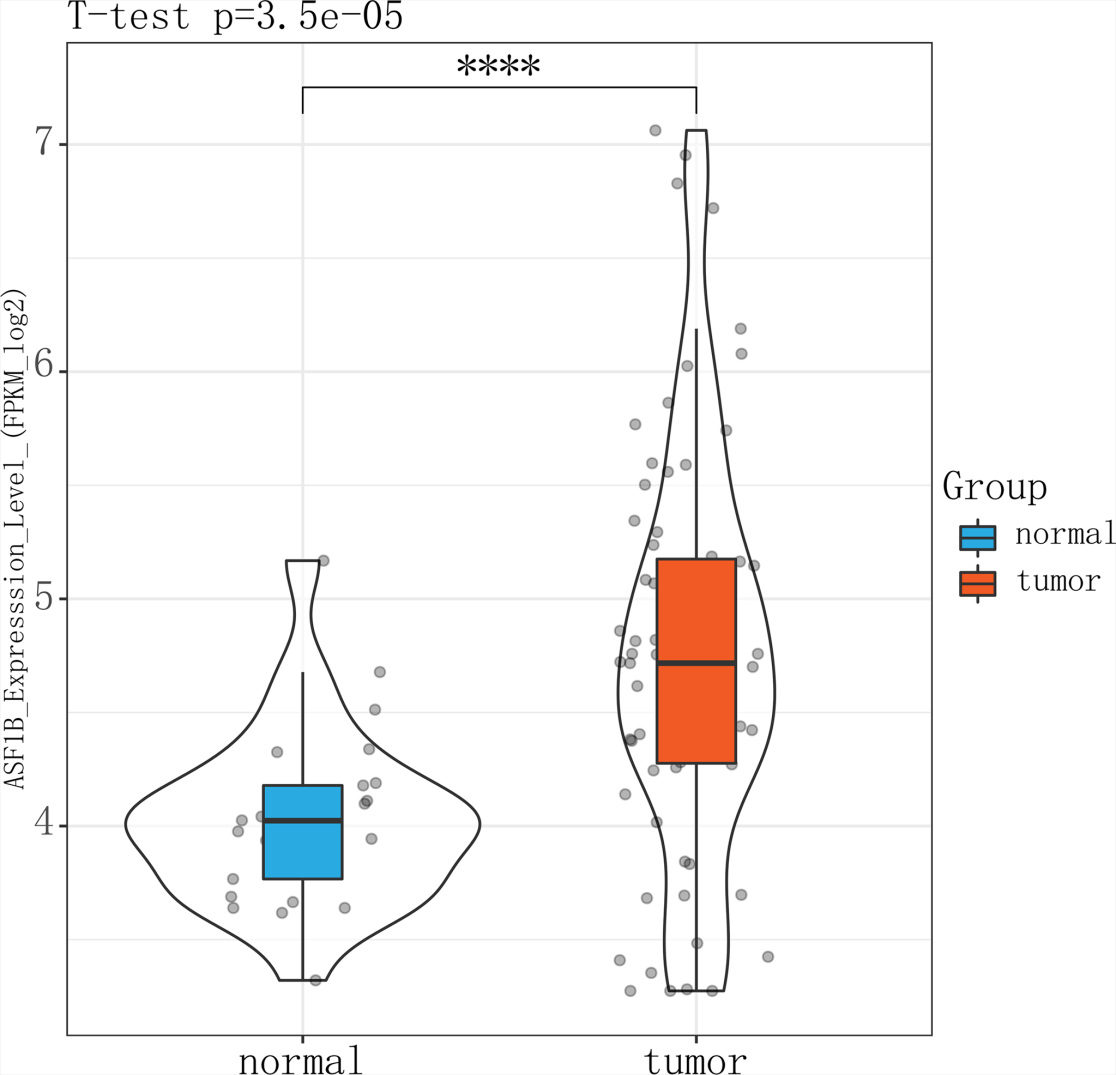
Violin plot showing that ASFB1 expression was higher in male TC patient tumor tissues than in normal thyroid tissues (independent samples t-test, ****P<0.01).

The high expression of ASF1B in male TC tissues was verified in an independent male TC dataset composed of the GSE-29265, GSE-3467, and GSE-76039 datasets (independent samples *t*-test, *p* ≤ 0.01) (**Figure 9**). These datasets were corrected for batch effects using the Combat method (**Supplementary Figure 4**).

### ASF1B Silencing Repressed Male TC Cell Growth and Metastasis *In Vitro*

**Figure 10:**
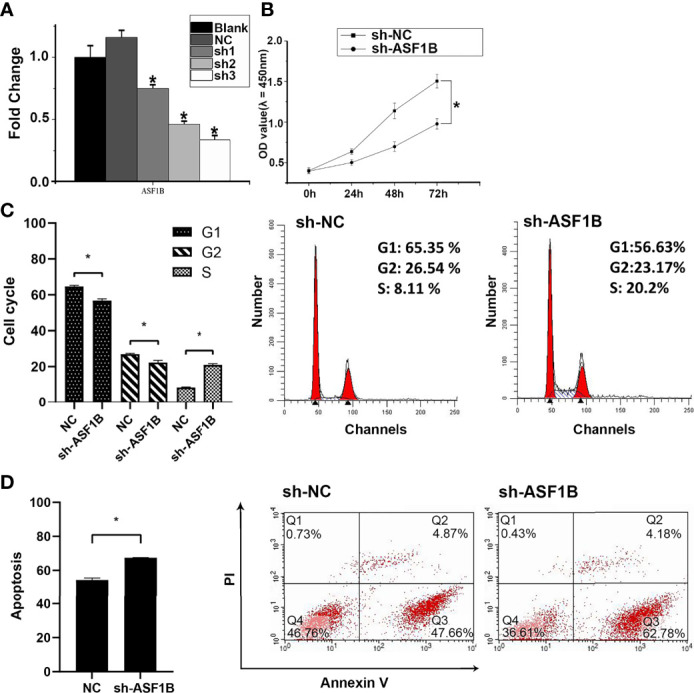
ASF1B could promote proliferation and inhibit apoptosis of male TC cells and accelerate cell cycle. **(A)** The knockdown efficiency of ASFB1 in KTC-1 cells was measured by real-time PCR. sh-ASF1B-3 (sh3) showed the best silencing effect and was used for the subsequent experiments, *P < 0.05. **(B)** Cell proliferation were detected by CCK-8 assays in KTC-1 cells, *P < 0.05. Detection of cell cycle **(C)** and cell apoptosis **(D)** in KTC-1 cells after transfection with sh-NC or sh-ASF1B by flow cytometry, respectively, *P < 0.05.

**Figure 11:**
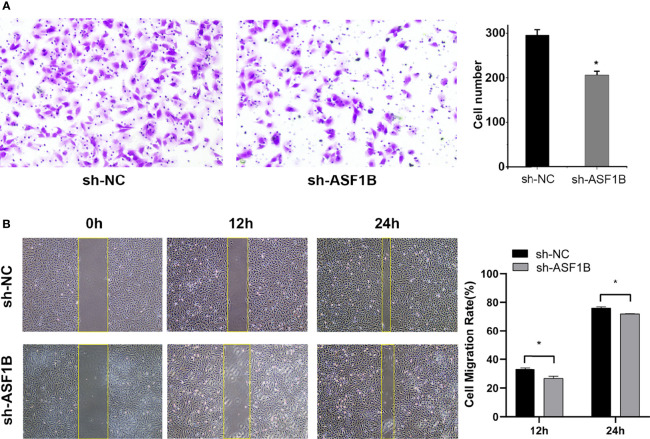
Knockdown of ASF1B inhibits male TC cell metastasis in intro. **(A)** Cell invasiveness was determined by Transwell assay in KTC-1 cells after transfection with sh-NC or sh-ASF1B, *P < 0.05. **(B)** Cell migration activity was performed by wound healing assay in KTC-1 cells after transfection with sh-NC or ASF1B, *P < 0.05.

Three sh-RNAs were designed to silence ASF1B, and sh-ASF1B-3 (sh3) showed the best silencing effect and was used for the subsequent experiments (**Figure 10A**). CCK8 assays showed that KTC-1 cell proliferation was significantly decreased in the sh-ASF1B group than the sh-NC group, *p* < 0.05 (**Figure 10B**). Apoptosis assays by flow cytometry indicated that ASF1B silence increased apoptosis rate in KTC-1 cells, *p* < 0.05 (**Figure 10D**). Meanwhile, more cells were found to be arrested in S phase in sh-ASF1B cell lines as well, *p* < 0.05 (**Figure 10C**). Additionally, wound healing assay and Transwell assay were applied to evaluate male TC cell migration and invasion, respectively. We found that compared with sh-NC group, the migrated cells (**Figure 11B**) and the invasive cells (**Figure 11A**) in the sh-ASF1B group decreased significantly, *p* < 0.05. These results suggested that ASF1B silencing inhibited male TC cell proliferation, cell cycle progression, migration, and invasion, and induced cell apoptosis.

### ASF1B Silencing Downregulated the Expression of p-AKT, AKT, and FOXP3 in Male TC Cells

**Figure 12:**
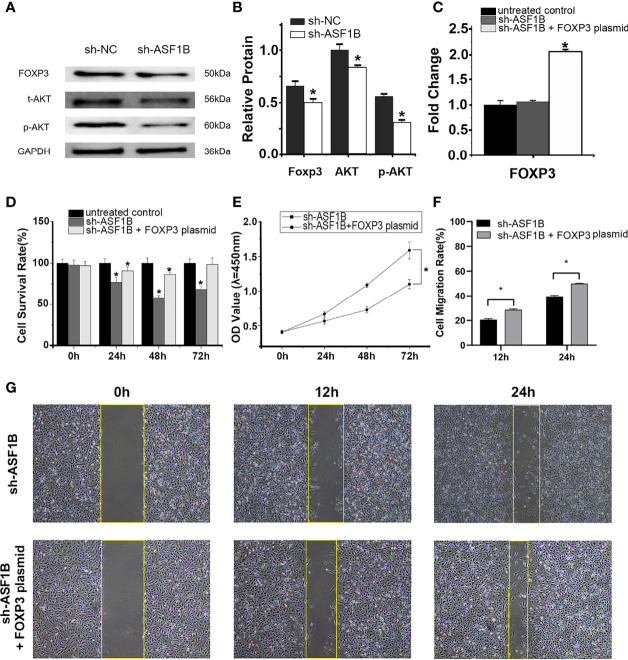
ASF1B targets p-AKT, AKT, and FOXP3, and rescue assays confirm that FOXP3 is an important downstream effector of ASF1B. Western blot analyses **(A)** and relative protein level test **(B)** indicated that ASF1B silencing decreased p-AKT, AKT, and FOXP3 protein levels, *p < 0.05. **(C)** RT-qPCR confirmed the overexpression of FOXP3 in FOXP3 overexpressing plasmid-transfected group, *p < 0.05. **(D)** Cell proliferation was detected by CCK-8 assays for three separate treatments, *p < 0.05. **(D)** The effect of FOXP3 overexpression on in ASF1B-silenced KTC-1 cell proliferation was determined by CCK-8 assay, *p < 0.05. **(F, G)** Cell migration activity was performed by wound healing assay in ASF1B-silenced KTC-1 cells after transfection with FOXP3-overexpressing plasmid or empty plasmid, *p < 0.05.

RT-qPCR and Western blot results showed that the silencing of ASF1B led to an increase in the mRNA and protein levels of p-AKT, AKT, and FOXP3 (**Figures 12A, B**). The results suggested that ASF1B might activate the PI3K/Akt signaling pathway through upregulating the p-Akt and Akt in male TC cells. FOXP3 also acts as a target of ASF1B, which could be involved in tumor immune escape and tumor cell proliferation.

### FOXP3 Is a Downstream Effector of ASF1B and Plays an Important Role in Male TC Cell Proliferation and Migration

RT-qPCR confirmed the overexpression of FOXP3 in the FOXP3-overexpressing plasmid-transfected group (**Figure 12C**; *p* < 0.05). In the scratch-wound assay, the width of the scratch was smaller in the FOXP3-overexpressing sh-ASF1B group than in the sh-ASF1B group (**Figures 12F, G**; *p* < 0.05). CCK8 assay showed that the FOXP3-overexpressing sh-ASF1B group had a significant higher proliferative ability than the sh-ASF1B group (**Figure 12E**; *p* < 0.05) and there was no significant difference between the FOXP3-overexpressing sh-ASF1B group and the untreated control group after a 72-h culture (**Figure 12D**; *p* > 0.05). These results suggest that the ability to migrate and proliferate was restored in ASF1B-silenced KTC-1 cells after being transfected with FOXP3-overexpressing plasmid.

## Discussion

The number of normal tissues was limited in the TCGA database, so we combined the data from the GTEx and TCGA databases. The large sample size increased the statistical power and increased the validity of our results. A total of 47 hub genes were identified with a series of bioinformatics analyses of these samples. Of these hub genes, only KCNQ3 had been previously reported in TC (45, 46). Most of the hub genes we identified have not been previously reported.

In WCGNA, one module consists of a specific group of genes. We identified two modules that were strongly correlated with lymphatic metastasis and tumor size (T stage) by WGCNA. Then, GO and KEGG enrichment analyses showed that the dominant function of the two modules was concentrated in cell membrane structure and composition. The results of previous studies have confirmed that cell membrane structure and composition components, such as glycans and phospholipids, play an important role in tumor initiation, development, metastasis, and chemoresistance (47–49). The sodium iodide symporter (NIS), which mediates active iodide transport into thyroid follicular cells, is a plasma membrane glycoprotein. A loss of NIS function has been observed in some TC patients. These patients have tumors that no longer trap iodine and hence are refractory to radioactive iodine (RAI), heralding a poor prognosis (50). Therefore, it is possible that cell membrane structure and composition are related to TC progression in males. However, there is a lack of evidence from *in vitro* or *in vivo* experiments to support this hypothesis.

ASF1B is a histone chaperone that is crucial for DNA replication and repair and transcriptional regulation (51). Corpet et al. demonstrated the ability of ASF1B to promote the proliferation of cells (52). It has been suggested that ASF1B overexpression promotes tumor progression and metastasis in several cancers, such as breast cancer (52), cervical cancer (53), clear cell renal cell carcinoma (54), and prostate cancer (55). By performing survival analyses of 47 hub genes using the univariate Cox analysis model, we demonstrated that male TC patients with high levels of ASF1B expression have a poor prognosis. Furthermore, ROC curve analysis showed that ASF1B had excellent predictive value for the DFS rate of male TC patients. These results are in accordance with those of recent studies indicating that ASF1B might play a crucial role in male TC progression. Our functional cellular experiments indicated that ASF1B silencing inhibited the proliferative and invasive capacities of KTC-1 cells, which were originally derived from male TC patients (43). Moreover, the silencing of ASF1B led to the downregulation of AKT and FOXP3 expression, suggesting a positive association between ASF1B and the PI3K/AKT pathway and between ASF1B and FOXP3. The PI3K/AKT pathway is commonly accepted as a vital pathway that controls cancer progression (56). Cunha et al. (57) also indicated that highly invasive TC cells had stronger nuclear FOXP3 staining than less aggressive TC cells. The results of another study demonstrated that the inhibition of FOXP3 in TC cells suppressed their proliferation and migration but promoted apoptosis (58). These results suggest that ASF1B may exert tumor-promoting effects on male TC cells through the PI3K/AKT pathway and FOXP3, which leads to a poor prognosis in male TC patients.

To obtain further insight into the role of ASF1B in TC in males, we further studied the immune-related mechanism of ASF1B using bioinformatics tools. We found a mild but significant positive correlation between ASF1B expression level and immune indicators (ESTIMATE score, immune score and Treg infiltration level). There are two possible reasons for this phenomenon (1): ASF1B indirectly regulates Treg infiltration level through an unknown intermediate regulatory factor (such as FOXP3), and (2) the comparatively small number of cases could add to the insufficiency of the significant differences. A previous study revealed that pancreatic cancer cells expressing high FOXP3 levels promoted the level of Treg infiltration through the secretion of TGF-h (29). Gogali et al. (59) reported that the density of Tregs in TC samples was higher than that in nodular goiter samples and was positively correlated with disease stage. In two other studies, Tregs were considered closely correlated with more aggressive TC (60, 61). Thus, we speculated that ASF1B may mediate Treg cells recruitment by regulating the expression of FOXP3. This may be one of the mechanisms of the immune escape of male TC cells.

Because of the lack of clinical evidence of the efficacy of ASF1B-targeted immunotherapy for TC in males, we used data from previously published urothelial cancer research to investigate the potential effect of ASF1B expression levels on the immunotherapy response. The results showed that the expression level of ASF1B was negatively correlated with the response rate to anti-PD-L1 therapy. This implied that male TC patients with high expression levels of ASF1B might have limited or no benefit from anti-PD-L1 therapy. Determining ASF1B expression levels could be considered a high priority for predicting the efficacy of PD-L1 targeted therapy.

Two recent studies show a role for androgens in PD-L1 expression (62) and cell proliferation (63). Both show evidence of a protective role for androgen signaling in thyroid cancer. However, these studies have some shortcomings. First, their experiments were performed in the 8505c cell line. It is a human undifferentiated TC cell line. Undifferentiated TC is a rare subtype of TC and accounts for only about 5% of all cases (64); it is unresponsive to conventional treatments such as radiotherapy or chemotherapy (65). Patients with undifferentiated thyroid cancer have been reported to have a poorer prognosis than DTC patients (64). Therefore, it is inappropriate to extrapolate the results of experiments performed in the 8505c cell line to all TC cell lines. Second, the 8505C cell line was established from TCs of a female patient (66). It is unreasonable to perform androgen-related experiments on a female cell line that aim to determine whether androgen is beneficial for male TC patients.

There are some limitations to our research. Firstly, only males were included in the study; it remains to be investigated whether ASF1B plays a similarly important role in female TC patients. Due to limited research funds, we did not conduct an in-depth study of the molecular mechanisms linking ASF1B to the progression of male TC. In addition, *in vivo* animal experiments must be further conducted to verify the results of the present study. Studies on the association between ASF1B and clinical outcomes of immune therapy are still lacking. We will continue to explore these areas in depth in the next stage of the study.

## Conclusion

In summary, we explored the hub genes and pathways that might be related to the progression and metastasis of TC in males by bioinformatics methods and cell experiments. Our results indicate that ASF1B could be considered a prognostic marker, therapeutic target, and predictor of immunotherapy response in male thyroid cancer patients. However, further studies are needed to investigate these issues.

## Data Availability Statement

The datasets presented in this study can be found in online repositories. The names of the repository/repositories and accession number(s) can be found in the article/**Supplementary Material**.

## Author Contributions

WQ and XW conceived and designed the study. HS, LL, and BL performed literature search. XW analyzed the data and generated the figures and tables. WQ wrote the manuscript and XW critically reviewed the manuscript. XW, WW, and JL supervised the research. All authors contributed to the article and approved the submitted version.

## Conflict of Interest

The authors declare that the research was conducted in the absence of any commercial or financial relationships that could be construed as a potential conflict of interest.

## Publisher’s Note

All claims expressed in this article are solely those of the authors and do not necessarily represent those of their affiliated organizations, or those of the publisher, the editors and the reviewers. Any product that may be evaluated in this article, or claim that may be made by its manufacturer, is not guaranteed or endorsed by the publisher.

## Supplementary Material

Supplementary Figure 1 Batch correction of RNA-seq data from the TCGA and GTEx databases.

Supplementary Figure 2 Determination of soft-thresholding power in weighted gene coexpression network analysis and gene clustering dendrograms. **(A)** Analysis of the scale-free topology model fit index for soft threshold powers. Horizontal axis is soft threshold power (β). Line indicates the threshold(β=4). **(B, C)** When soft threshold power (β) = 4, the mean connectivity was almost 0.

Supplementary Figure 3 Venn diagrams showing the overlapping hub genes between the weighted gene coexpression network and the PPI networks.

Supplementary Figure 4 Results of Gene Ontology (GO) and Kyoto Encyclopedia of Genes and Genomes (KEGG) enrichment analyses for the gene sets of the modules containing hub genes. BP, biological process; CC, cellular component; MF, molecular function. In each histogram, light blue represents the BP pathway, green represents the CC pathway, deep blue represents the MF pathway, and purple represents the KEGG pathway. The name of each module is marked above each histogram. The height of the histogram bar corresponds to the number of genes enriched in each pathway. The name of the pathway is labeled on the bottom of the histogram bar. The orange broken line represents the enrichment score of each pathway. We obtained these scores by taking the negative log10 of the p-values of the enrichment analysis of each pathway. The left y-axis represents the number of genes. The right y-axis represents the enrichment score.

Supplementary Figure 5 Batch correction of RNA-seq data from 3 GEO datasets.

Supplementary Table 1 Clinical and follow-up data of male TC patients from the TCGA database.

Supplementary Table 2 Differentially expressed genes in male TC patients.

Supplementary Table 3 Hub genes screened from differentially expressed genes by weighted gene coexpression network analysis.

Supplementary Table 4 Hub genes screened from the protein–protein interaction network.

Supplementary Table 5 Survival analysis of hub genes.

Supplementary Table 6 shRNA sequences.

Supplementary Table 7 FOXP3-overexpressing plasmid sequences.
