# Supplementary figures and images for: ASF1B: A Possible Prognostic Marker, Therapeutic Target, and Predictor of Immunotherapy in Male Thyroid Carcinoma
Source: Front Oncol. 2022 Jan 31;12:678025. doi: 10.3389/fonc.2022.678025 (PMC8841667; doi:10.3389/fonc.2022.678025)

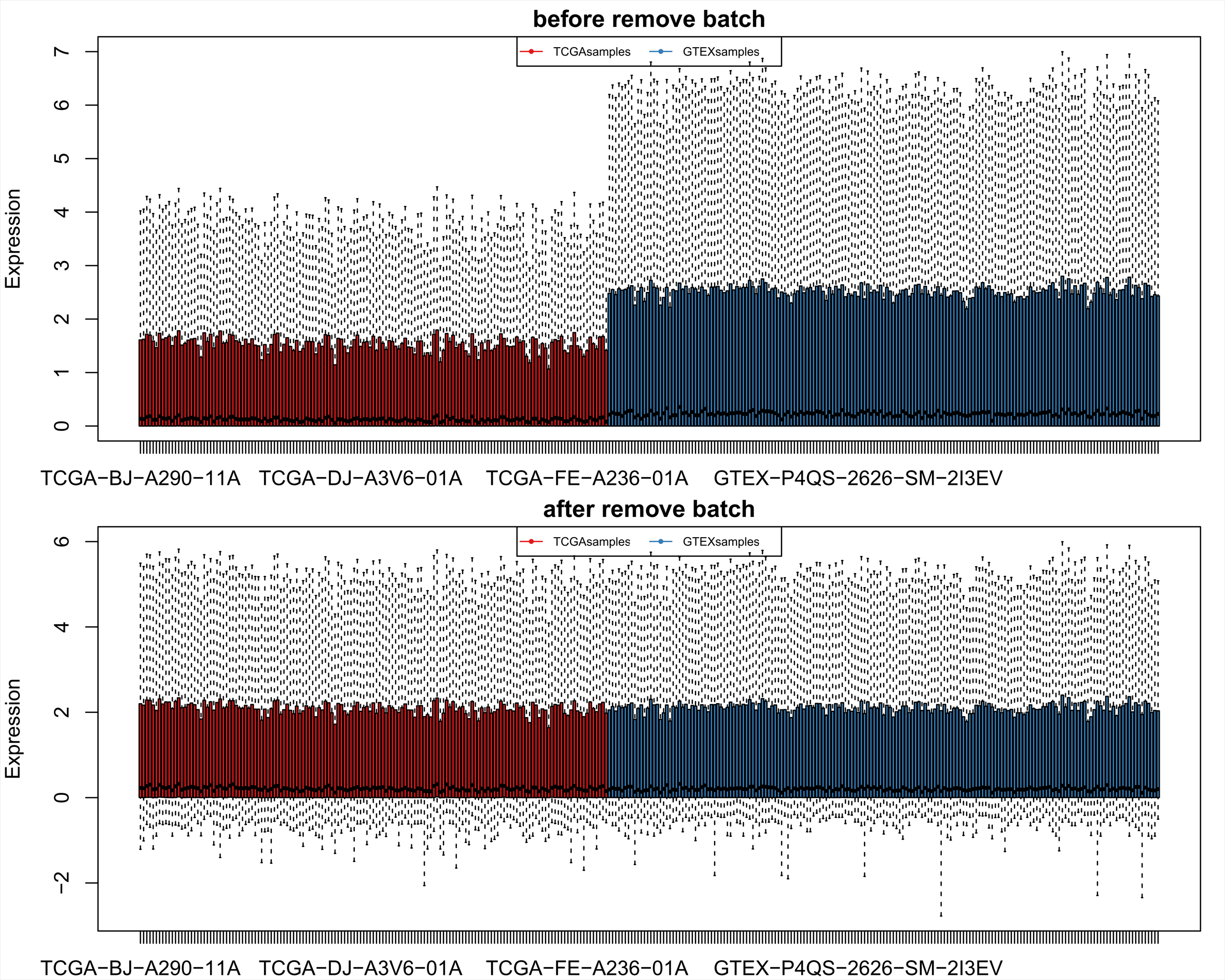

Supplement: Supplementary Figure 1 — Batch correction of RNA-seq data from the TCGA and GTEx databases. [file Image_1.tif]

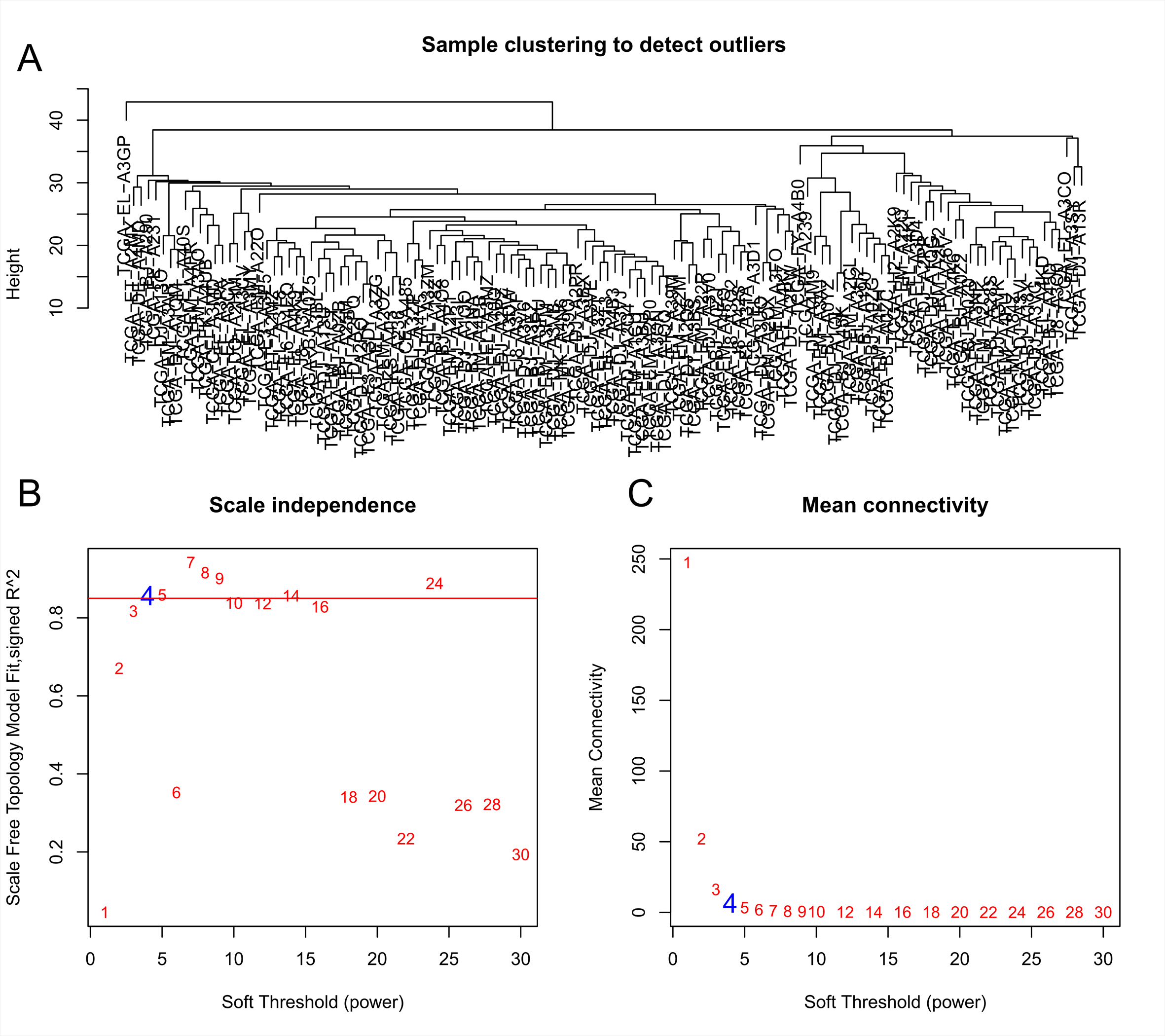

Supplement: Supplementary Figure 2 — Determination of soft-thresholding power in weighted gene coexpression network analysis and gene clustering dendrograms. (A) Analysis of the scale-free topology model fit index for soft threshold powers. Horizontal axis is soft threshold power (β). Line indicates the threshold(β=4). (B, C) When soft threshold power (β) = 4, the mean connectivity was almost 0. [file Image_2.tif]

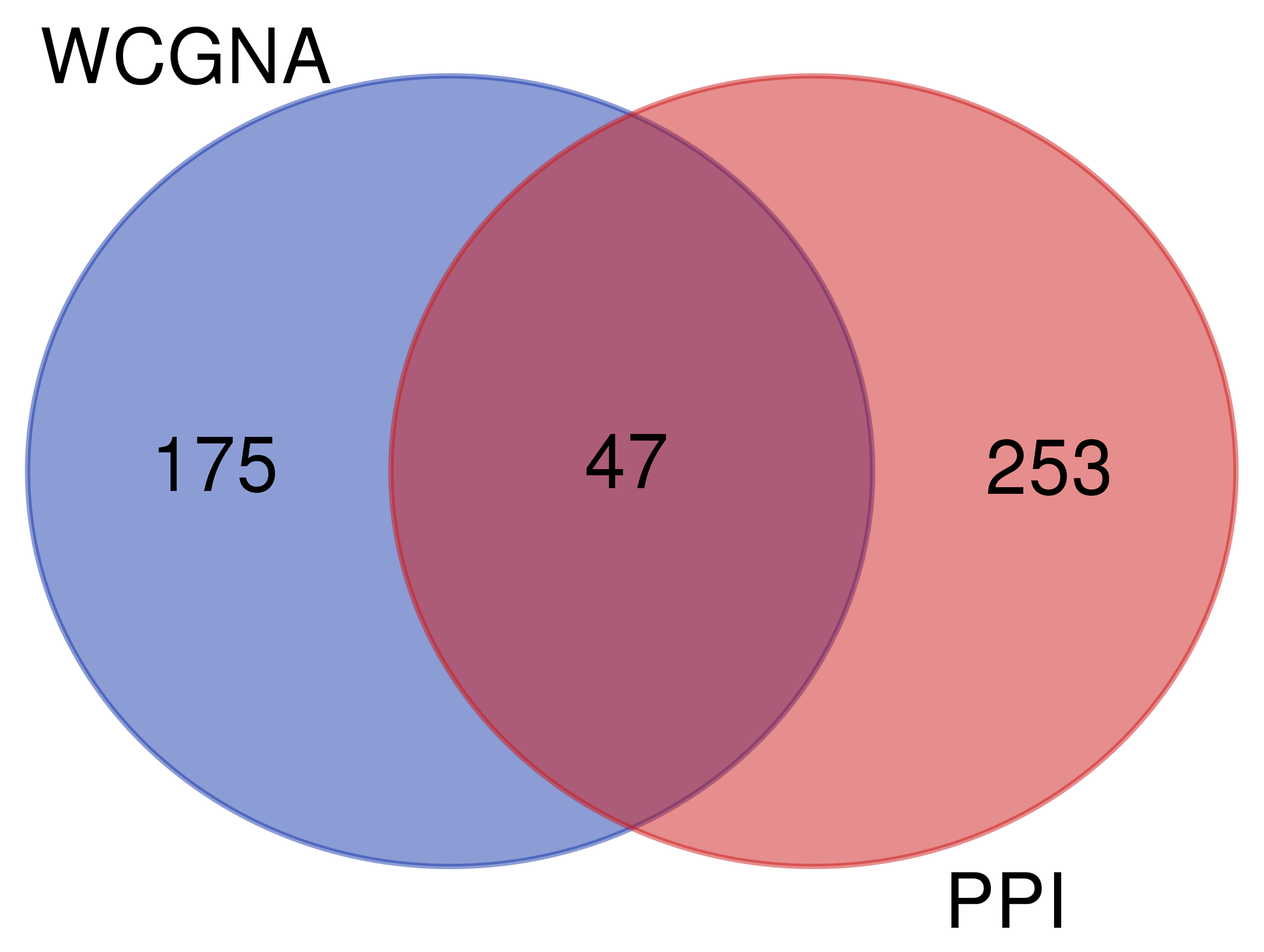

Supplement: Supplementary Figure 3 — Venn diagrams showing the overlapping hub genes between the weighted gene coexpression network and the PPI networks. [file Image_3.tiff]

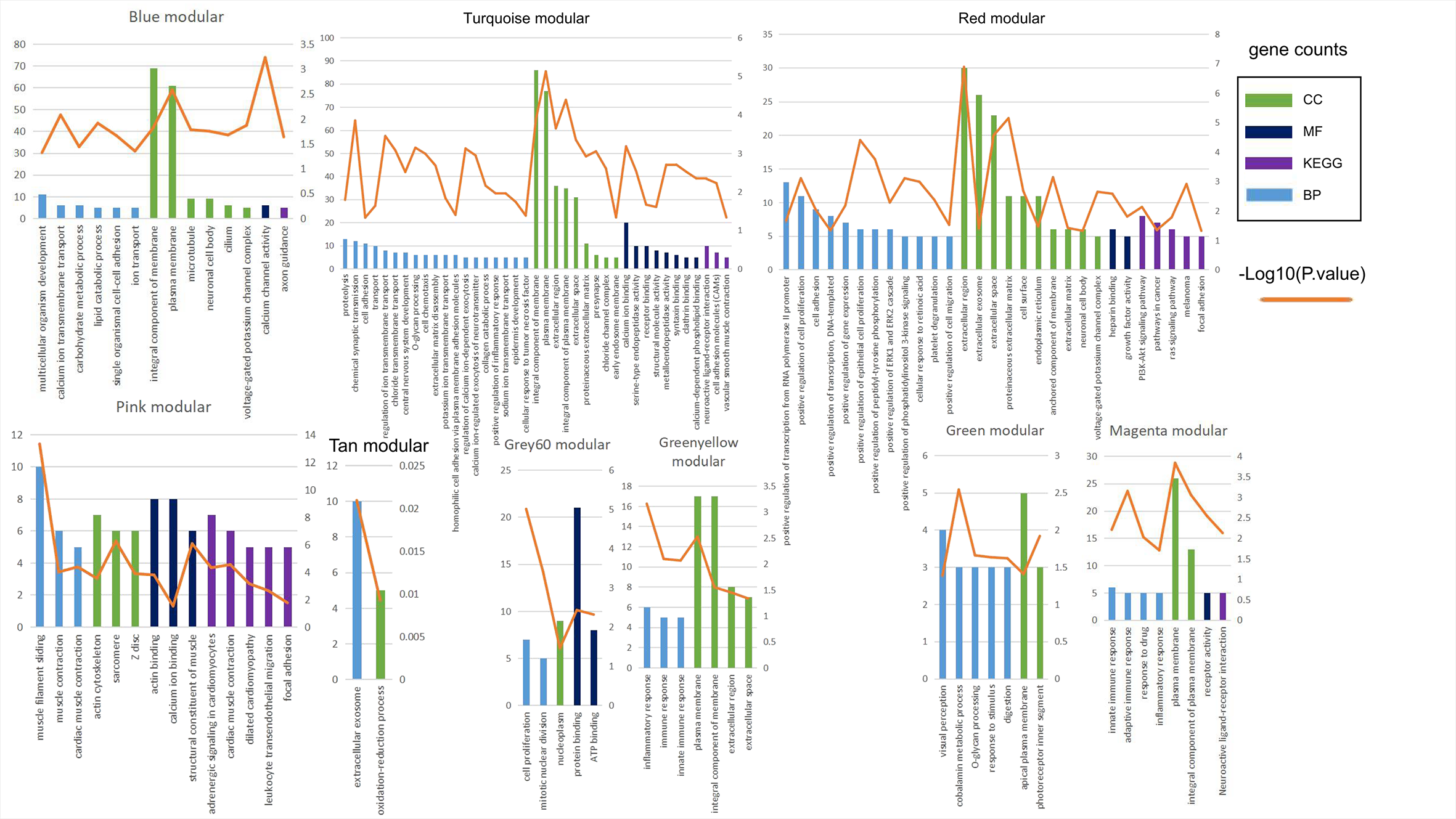

Supplement: Supplementary Figure 4 — Results of Gene Ontology (GO) and Kyoto Encyclopedia of Genes and Genomes (KEGG) enrichment analyses for the gene sets of the modules containing hub genes. BP, biological process; CC, cellular component; MF, molecular function. In each histogram, light blue represents the BP pathway, green represents the CC pathway, deep blue represents the MF pathway, and purple represents the KEGG pathway. The name of each module is marked above each histogram. The height of the histogram bar corresponds to the number of genes enriched in each pathway. The name of the pathway is labeled on the bottom of the histogram bar. The orange broken line represents the enrichment score of each pathway. We obtained these scores by taking the negative log10 of the p-values of the enrichment analysis of each pathway. The left y-axis represents the number of genes. The right y-axis represents the enrichment score. [file Image_4.tif]

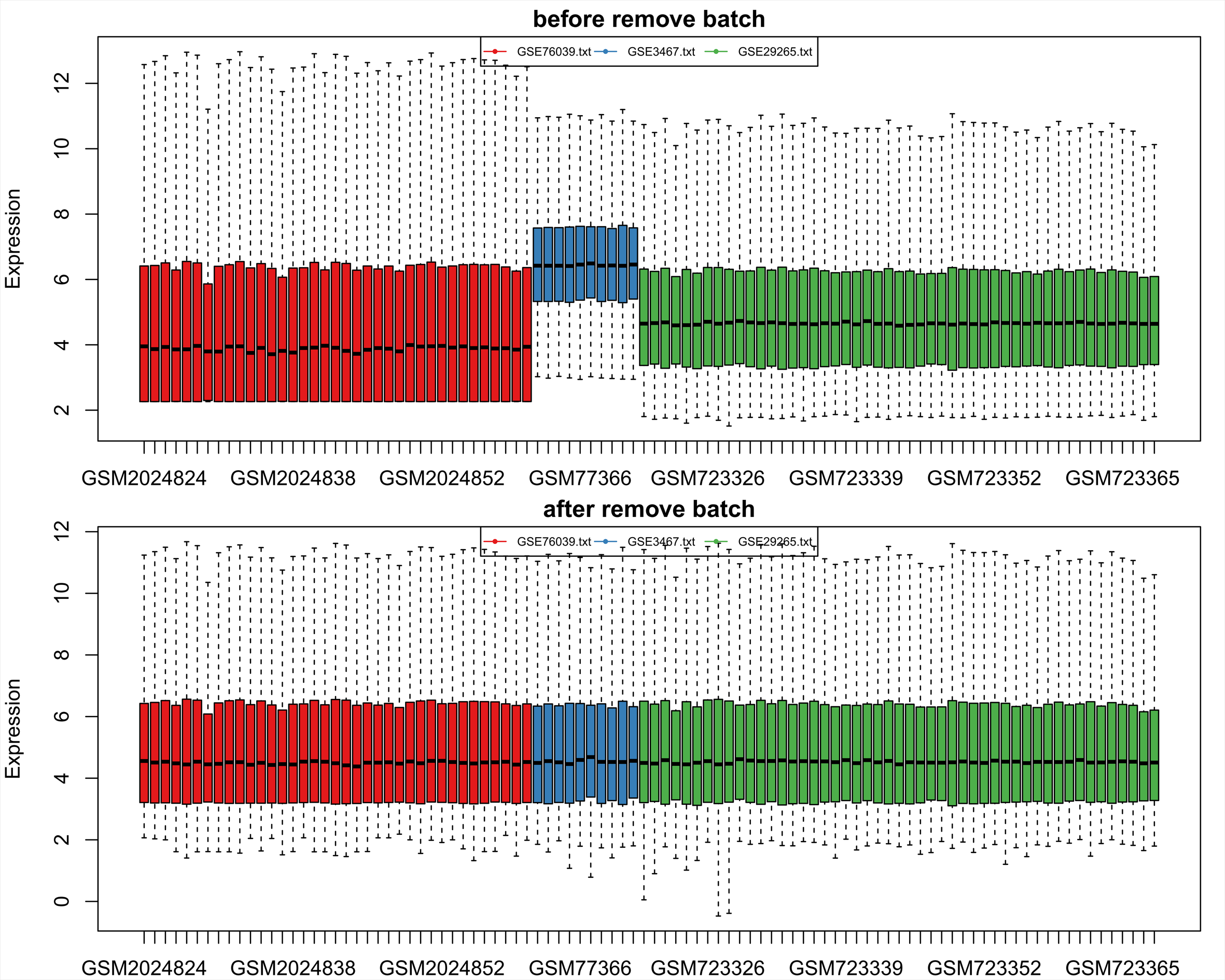

Supplement: Supplementary Figure 5 — Batch correction of RNA-seq data from 3 GEO datasets. [file Image_5.tif]
