# Supplementary material for: ASF1B: A Possible Prognostic Marker, Therapeutic Target, and Predictor of Immunotherapy in Male Thyroid Carcinoma
Source: Front Oncol. 2022 Jan 31;12:678025. doi: 10.3389/fonc.2022.678025 (PMC8841667; doi:10.3389/fonc.2022.678025)
Supplement: Supplementary Table 7 — FOXP3-overexpressing plasmid sequences. [file Table_7.doc]

**FOXP3-overexpressing plasmid**

**Gene Sequence：**

//TCTAGAATGCCCAACCCCAGGCCTGGCAAGCCCTCGGCCCCTTCCTTGGCCCTTGGCCCATCCCCAGGAGCCTCGCCCAGCTGGAGGGCTGCACCCAAAGCCTCAGACCTGCTGGGGGCCCGGGGCCCAGGGGGAACCTTCCAGGGCCGAGATCTTCGAGGCGGGGCCCATGCCTCCTCTTCTTCCTTGAACCCCATGCCACCATCGCAGCTGCAGCTGCCCACACTGCCCCTAGTCATGGTGGCACCCTCCGGGGCACGGCTGGGCCCCTTGCCCCACTTACAGGCACTCCTCCAGGACAGGCCACATTTCATGCACCAGCTCTCAACGGTGGATGCCCACGCCCGGACCCCTGTGCTGCAGGTGCACCCCCTGGAGAGCCCAGCCATGATCAGCCTCACACCACCCACCACCGCCACTGGGGTCTTCTCCCTCAAGGCCCGGCCTGGCCTCCCACCTGGGATCAACGTGGCCAGCCTGGAATGGGTGTCCAGGGAGCCGGCACTGCTCTGCACCTTCCCAAATCCCAGTGCACCCAGGAAGGACAGCACCCTTTCGGCTGTGCCCCAGAGCTCCTACCCACTGCTGGCAAATGGTGTCTGCAAGTGGCCCGGATGTGAGAAGGTCTTCGAAGAGCCAGAGGACTTCCTCAAGCACTGCCAGGCGGACCATCTTCTGGATGAGAAGGGCAGGGCACAATGTCTCCTCCAGAGAGAGATGGTACAGTCTCTGGAGCAGCAGCTGGTGCTGGAGAAGGAGAAGCTGAGTGCCATGCAGGCCCACCTGGCTGGGAAAATGGCACTGACCAAGGCTTCATCTGTGGCATCATCCGACAAGGGCTCCTGCTGCATCGTAGCTGCTGGCAGCCAAGGCCCTGTCGTCCCAGCCTGGTCTGGCCCCCGGGAGGCCCCTGACAGCCTGTTTGCTGTCCGGAGGCACCTGTGGGGTAGCCATGGAAACAGCACATTCCCAGAGTTCCTCCACAACATGGACTACTTCAAGTTCCACAACATGCGACCCCCTTTCACCTACGCCACGCTCATCCGCTGGGCCATCCTGGAGGCTCCAGAGAAGCAGCGGACACTCAATGAGATCTACCACTGGTTCACACGCATGTTTGCCTTCTTCAGAAACCATCCTGCCACCTGGAAGAACGCCATCCGCCACAACCTGAGTCTGCACAAGTGCTTTGTGCGGGTGGAGAGCGAGAAGGGGGCTGTGTGGACCGTGGATGAGCTGGAGTTCCGCAAGAAACGGAGCCAGAGGCCCAGCAGGTGTTCCAACCCTACACCTGGCCCCCGCGGCCGC//
